# In vitro evaluation of cerebrospinal fluid velocity measurement in type I Chiari malformation: repeatability, reproducibility, and agreement using 2D phase contrast and 4D flow MRI

**DOI:** 10.1186/s12987-021-00246-3

**Published:** 2021-03-18

**Authors:** Gwendolyn Williams, Suraj Thyagaraj, Audrey Fu, John Oshinski, Daniel Giese, Alexander C. Bunck, Eleonora Fornari, Francesco Santini, Mark Luciano, Francis Loth, Bryn A. Martin

**Affiliations:** 1 Department of Chemical and Biological Engineering, University of Idaho, 875 Perimeter Dr. MC1122, Moscow, ID 83844 USA; 2 Department of Mechanical Engineering, Conquer Chiari Research Center, University of Akron, Akron, OH 44325 USA; 3 Department of Mathematics and Statistical Science, University of Idaho, Moscow, ID 83844 USA; 4 Department of Radiology and Imaging Sciences, Emory University, Atlanta, GA 30322 USA; 5 Department of Radiology, University Hospital of Cologne, Cologne, Germany; 6 CIBM, Department of Radiology, Centre Hospitalier Universitaire Vaudois (CHUV) and University of Lausanne, Lausanne, Switzerland; 7 Division of Radiological Physics, Department of Radiology, University Hospital of Basel, Basel, Switzerland; 8 Department of Biomedical Engineering, University of Basel, Allschwil, Switzerland; 9 Department of Neurosurgery, John Hopkins University, Baltimore, MD USA; 10 Alcyone Therapeutics Inc, Lowell, MA USA

**Keywords:** Cerebrospinal fluid, Phase contrast, Magnetic resonance imaging, Chiari malformation, Spinal cord

## Abstract

**Background:**

Phase contrast magnetic resonance imaging, PC MRI, is a valuable tool allowing for non-invasive quantification of CSF dynamics, but has lacked adoption in clinical practice for Chiari malformation diagnostics. To improve these diagnostic practices, a better understanding of PC MRI based measurement agreement, repeatability, and reproducibility of CSF dynamics is needed.

**Methods:**

An anatomically realistic in vitro subject specific model of a Chiari malformation patient was scanned three times at five different scanning centers using 2D PC MRI and 4D Flow techniques to quantify intra-scanner repeatability, inter-scanner reproducibility, and agreement between imaging modalities. Peak systolic CSF velocities were measured at nine axial planes using 2D PC MRI, which were then compared to 4D Flow peak systolic velocity measurements extracted at those exact axial positions along the model.

**Results:**

Comparison of measurement results showed good overall agreement of CSF velocity detection between 2D PC MRI and 4D Flow (p = 0.86), fair intra-scanner repeatability (confidence intervals ± 1.5 cm/s), and poor inter-scanner reproducibility. On average, 4D Flow measurements had a larger variability than 2D PC MRI measurements (standard deviations 1.83 and 1.04 cm/s, respectively).

**Conclusion:**

Agreement, repeatability, and reproducibility of 2D PC MRI and 4D Flow detection of peak CSF velocities was quantified using a patient-specific in vitro model of Chiari malformation. In combination, the greatest factor leading to measurement inconsistency was determined to be a lack of reproducibility between different MRI centers. Overall, these findings may help lead to better understanding for application of 2D PC MRI and 4D Flow techniques as diagnostic tools for CSF dynamics quantification in Chiari malformation and related diseases.

**Supplementary Information:**

The online version contains supplementary material available at 10.1186/s12987-021-00246-3.

## Introduction

The dynamic movement of cerebrospinal fluid (CSF) has long been the subject of scientific investigation, and its important functional role to support central nervous system health is increasingly realized. For this reason, non-invasive phase contrast magnetic resonance imaging (PC MRI) quantification of CSF dynamics has been pursued for diagnosis, prognosis, and treatment of neurological diseases such as hydrocephalus [1, 2], Chiari malformation [3], and syringomyelia [4, 5]. Variabilities in CSF dynamics, such as increased CSF velocities and/or flow rate, are thought to be indicative of Chiari malformation and related neurological disorders [6, 7]. Single-plane two-dimensional, through-plane encoded PC MRI (2D PC MRI) and time-resolved three-dimensional velocity encoded PC MRI (4D Flow) are promising modalities that allow for CSF dynamics characterization. 2D PC MRI is one of the best known non-invasive methods and currently the only method for both qualitative and quantitative CSF characterization [8]. Clinical application of 2D PC MRI is widely varied with use in visualizing morphological and functional alterations in normal pressure hydrocephalous patients as well as CSF flow assessment in Chiari malformation populations with and without syringomyelia [9]. 4D Flow has shown potential to advance in vivo assessment of complex hemodynamic and CSF flow patterns [10–12]. Originally developed for cardiovascular applications [13], 4D Flow has been applied to analyze CSF velocity differences between healthy controls and Chiari malformation patients, with and without syrinx formation [14]. Contrast-enhanced MRI techniques have also been applied to quantify relatively slow timescale transport phenomena, such as CSF solute transport in humans [15–17]. Additionally, MRI has been applied to quantify short timescale phenomena such as dynamic motion of CSF due to respiration and other maneuvers using real-time PC MRI [18–21] and time-slip MRI [22, 23]. These methods show promise to help reveal new insights about CSF system physiology in health and disease.

At present, the diagnostic relevance of PC MRI-based measurement of CSF velocity dynamics remains under debate by the medical community. For example, the recently published National Institutes of Health common data elements (CDEs) for Chiari malformation clinical research does not include any recommended measurements related to CSF dynamics [24]. The lack of adoption of CSF dynamics as a standard measure for Chiari malformation is likely due to the conflicting findings reported in previous studies comparing CSF velocities in Chiari malformation patients and healthy controls [25–28]. For example, some investigators report elevated CSF velocities in Chiari malformation patients’ pre-surgical treatment, and others reported decreased pre-surgical CSF velocities in Chiari malformation patients compared to post-surgery. Also, there are conflicting reports of both elevated and decreased CSF velocities in healthy subjects compared to Chiari malformation patients. These conflicting findings were discussed in a review by Shaffer et al. [6].

To address the need for improved CSF dynamics quantification, the present study aims to quantify the agreement, reproducibility, and repeatability of 4D Flow and 2D PC MRI measurement of CSF velocities at the craniovertebral junction. Our focus was the craniovertebral junction CSF velocities because these velocities are thought to potentially be a diagnostic indicator of Chiari malformation. To mitigate normal physiological variation in CSF velocities, our approach utilized a subject-specific high-resolution 3D printed model of a Chiari malformation patient with computer controlled pulsatile CSF pump [29]. We hypothesized that 2D PC MRI and 4D Flow would have strong measurement agreement, repeatability, and reproducibility.

### Literature review

**Fig. 1:**
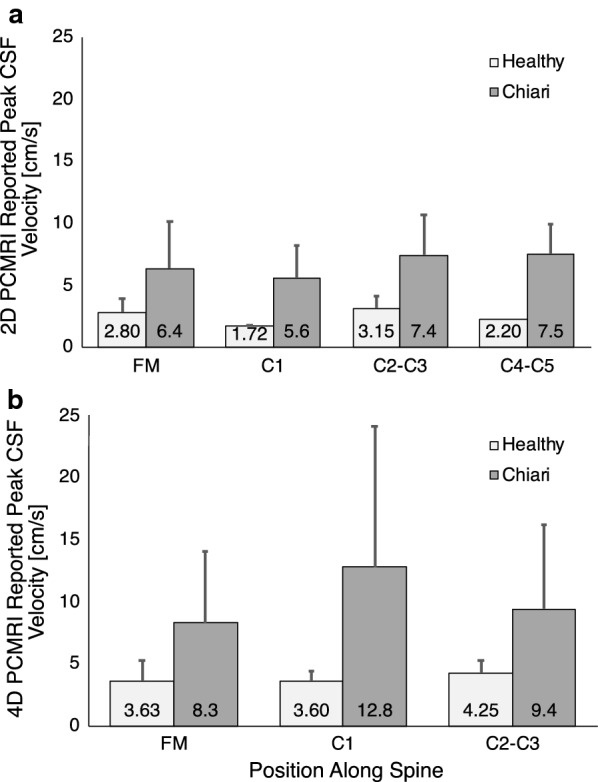
Summary of the average peak CSF velocities reported in 2D PC MRI (**a**) and 4D PC MRI (**b**) literature for healthy subjects and Chiari malformation patients pre-decompression surgery. Average values in figure are weighted by number of subjects within each study. Error bars represent pooled reported standard deviation for studies included in each group. The total number of healthy and Chiari malformation patient studies included is 91 and 166, respectively (see Table 1 for individual values). FM = foramen magnum

We conducted a meta-analysis of all CSF velocity quantification studies applied in Chiari malformation (Table 1). These studies show a range of peak CSF velocities in healthy controls and Chiari patients depending on the measurement position along the spine, voxel size, slice thickness, and number of phases. Figure 1 provides a summary of Table 1 results in terms of the average CSF velocities reported in the studies at each axial slice position along the spine (FM to C5) for healthy subjects (N = 91 included across all studies analyzed) and Chiari malformation patients that have not received decompression surgery (N = 166 included across all studies analyzed). Additional file 1: Fig. S1 contains Forest plots depicting the meta-analysis for each imaging methodology and treatment group, showing the spread of reported peak systolic CSF velocities. This meta-analysis shows peak CSF velocities are elevated in Chiari malformation compared to healthy subjects and the axial position of greatest CSF velocity elevation is most commonly reported at the FM - C1 vertebral level (Fig. 1). However, the standard deviation of peak CSF velocities is considerable compared to group differences and this variance makes specification of a diagnostic threshold for patients versus controls difficult. Notably, several studies included in the meta-analysis had Chiari cohorts with syringomyelia, which is known to affect CSF dynamics [14]. The comorbidity of Chiari and syringomyelia complicates the assessment of Chiari CSF dynamics and requires further investigation to accurately describe the contributions of Chiari and syringomyelia to the CSF dynamics.

**Table 1:** Literature review of 2D PC MRI (N = 208) and 4D PC MRI (N = 49) in vivo measurements of peak CSF velocities in healthy (H, N = 91) and Chiari malformation patient (P, N = 166) cases

Study	MR sequence	Subject (N) Healthy/Patient	Axial region	Peak reported velocity (cm/s)	In-plane resolution (mm)	Slice thickness (mm)	# of phases per cycle	Venc (cm/s)	MR Scanner
Bunck et al. [54]	4D PC MRI	H (10)	FM	3.6 ± 2.0	1.5	1.5	12–14	10,15	1.5 T Philips Achieva 2.6
			C1	3.6 ± 0.8					
			C2	4.5 ± 1.0					
		P (2)	C2/C3	19.7 ± 0.2					
Bunck et al. [14]	4D PC MRI	H (10)	FM	3.2 ± 1.0	1.5	1.5	12–14	20	1.5 T Philips Achieva 2.6
			C1	3.6 ± 0.8					
			C2	4.0 ± 1.0					
		P (20)	FM	7.6 ± 5.0					
			C1	12.8 ± 11.3					
			C2	8.4 ± 6.9					
Yiallourou et al. [55]	4D PC MRI	H (3)	FM	5.2 ± 1.8	1.5	1.5	N/R	10	1.5 T Philips Achieva 2.6
		P (4)	FM	11.8 ± 9.0				20	
Shah et al. [56]	2D PC MRI	P (17)	FM to C2	5.6 ± 2.6	0.7	5	14	10	N/R
			C4	7.5 ± 2.4					
Houghton et al. [57]	2D PC MRI	H (10)	FM	2.8 ± 1.0	0.7	5	14	10	1.5 T scanner
		P (8)	FM	4.0 ± 1.0					
Dolar et al. [58]	2D PC MRI	P (8)	FM	6.8 ± 5.1	0.7	5	14	10	N/R
Kruger et al. [9]	2D PC MRI	P (45)	FM	6.3 ± 4.0	0.7	5	14	10	1.5 T scanner
Hofmann et al. [59]	2D PC MRI	H (18)	C2/C3	3.1 ± 1.7*	0.7	5	16	10	1.5 T scanner
Iskandar et al. [60]	2D PC MRI	H (1)	FM	4.2	0.7	5	14	10	1.5 T scanner
		P (8)	FM	9.7 ± 2.3					
Rutkowska et al. [61]	2D PC MRI	P (3)	FM	7.5 ± 3.5	0.7	5	N/R	10	1.5 T scanner
Loth et al._ENREF_17 [62]	2D PC MRI	H (1)	C2/C3	4.0	0.7	5	N/R	3–15	1.5 T Signa, GE Medical Systems
Cheng et al. [63]	2D PC MRI	H (1)	C5	2.2*	N/R	5	N/R	N/R	3 T Philips Achieva TX
Alperin et al. [41]	2D PC MRI	H (37)	C2	1.72 ± 0.06	Anisotropic 0.56 × 0.6	5 – 6	32	7 – 8	3 T Magnetom Verio, Siemens
		P (36)	C2	1.61 ± 0.05					
Alperin et al. [64]	2D PC MRI	P (15)	C2	1.93 ± .78	0.56	5	N/R	7 – 8	3 T Magnetom Trio, Siemens

The peak velocities denoted by an asterisk were measured at points/probes and not throughout the axial plane. H* indicates healthy subjects with a syrinx

Reproducibility and repeatability of CSF velocity measurements, measured in cm/s for individual voxels collected for a region of interest at the craniocervical junction, have not been specifically investigated. A number of studies have been conducted on the reliability of arterial hemodynamics using 4D Flow [30] and 2D PC MRI [31, 32] measurements. However, arterial flow velocities are typically one order of magnitude greater than CSF velocities. Thus, the reproducibility/repeatability results from these arterial hemodynamics studies are difficult to apply for CSF velocities. Repeatability of 2D PC MRI CSF and cerebral blood flow (mm^3^/s) measurements have been investigated and shown to have moderate in vivo test–retest repeatability [33]. In that study, the authors did not quantify reliability of CSF velocity measurement (cm/s) that has been a focus of interest for CSF-based Chiari malformation diagnostic tests. Repeatability of in vivo 2D PC MRI measurements of CSF flow at the aqueduct of Sylvius has been examined and found to have moderate repeatability [33]. However, aqueductal CSF velocities are typically greater than at the craniocervical junction. Also, the CSF space geometry at the craniocervical junction is more complex than the tube-shaped aqueductal geometry. The craniocervical junction anatomy is an annulus shape that contains spinal cord nerve roots, neuroaxis curvature, and tonsillar descent in Chiari malformation patients. Poor 4D Flow accuracy has been found during timeframes corresponding to low CSF flow rate [34], but further research is necessary before clinical application is feasible.

While many studies have previously quantified repeatability and operator effects for PC MRI hemodynamic and cerebral blood flow characterization [35–42], few studies have quantified these parameters for PC MRI CSF dynamics characterization (Table 2). Overall, these previous CSF dynamics studies are stratified into focuses on the cerebral aqueduct, the spinal subarachnoid space (SAS), and the C2–C3 area and are summarized in Table 2. These studies consistently reported strong intra/inter operator agreement and peak velocity measurements are independent of the operator, therefore intra/inter-operator effects are null in this context and were not investigated. A study by Tawfik et al. [43] detailed 2D PC MRI measurement repeatability at the cerebral aqueduct and reported a peak velocity standard deviation of 1.9 cm/s, which is comparable to the 1.83 cm/s peak velocity standard deviation we found in the cervical spine (Table 2). In vivo studies by Sakhare et al. [33] and Luetmer et al. [44] reported standard deviations of 2D PC MRI CSF flow between 0.04 and 0.98 mL/s but did not look at peak velocity values. Pahlavian et al. [34] performed an accuracy study on 4D Flow quantification of CSF dynamics using a 3D printed in vitro model similar to the one used here and found fairly high accuracy (95% CI ± 1.8 cm/s, Table 2) but did not quantify repeatability nor reproducibility of measurements. These accuracy results from Pahlavian et al. were of similar range as the reproducibility results of this study. To our knowledge, this is the first study to specifically detail the agreement between 2D PC MRI and 4D Flow quantification of peak CSF velocities and characterize reproducibility of measurements across different scanners.

**Table 2:** Literature review of repeatability, reproducibility, and agreement studies of PC MRI fluid velocity quantification techniques

Location	Study	In vitro	In vivo	Protocol	Repeatability (STD, σ)	Reproducibility	Agreement	Intra-rater	Inter-rater	Accuracy
Cervical Spine	Williams et al.	x		2D PC MRI	1.04 cm/s	Table 5	Table 5			
				4D Flow	1.83 cm/s					
Cervical Spine	Pahlavian et al. [34]	x		4D Flow						± 1.8 cm/s 95% CI
Cerebral Aqueduct	Sakhare et al. [33]		x	2D PC MRI	0.042 mL/s			0.99 ICC	0.99 ICC	
Cerebral Aqueduct	Luetmer et al. [44]	x	x	2D PC MRI	0.291 mL/s			6.4% CV	5.4% CV	
Cerebral Aqueduct	Tawfik et al. [43]		x	2D PC MRI	1.9 cm/s			0.88 ICC	0.88 ICC	
SAS	Sakhare et al. [33]		x	2D PC MRI	0.981 mL/s			0.94 ICC	0.94 ICC	
Upper C2	Koerte et al. [65]		x	2D PC MRI				0.881 ICC	0.985 ICC	

The large variance in CSF velocities reported in Chiari malformation patients versus controls (Fig. 1) in literature is likely due to the wide range in PC MRI acquisition methods and post-processing techniques. Factors contributing to inconsistency in PC MRI measurement results can be summarized as follows: (1) human error introduced by operator region of interest selection and variance of measurement location particularly with 2D techniques [45], (2) inconsistency in eddy current offset correction [34], (3) spatial resolution of MRI slices [8], (4) temporal resolution of number of phases sampled per cardiac cycle [46], (5) transient impact of respiration on time-average CSF flow measured by PC MRI [18, 21, 22] (6) Orientation of the neck angulation [47], (7) normal physiological variance in CSF flow [48], (8) noise and other imaging artefacts generated from subject motion in the MRI scanner [34, 45, 47, 49], (9) respiration-induced B_0_ variations [50].

## Methods

### Study design

Experiments were performed using an in vitro subject-specific CSF flow model of a Chiari malformation patient that was tested at five different MRI scanners at four different scanning centers. The centers were physically located as follows: Center 1, University Hospital in Cologne Germany (3T Achieva, Philips Healthcare, Best, Netherlands); Center 2, Emory University in Atlanta, Georgia, U.S.A (Siemens 3T PrismaFit, Atlanta, Georgia, U.S.A); Center 4, University Hospital in Basel Switzerland (3T, MAGNETOM Prisma, Siemens Healthcare, Erlangen, Germany); Centers 3 and 5 were both located at University Hospital in Lausanne Switzerland (3T PrismaFit and 3T Tim Trio, respectively, Siemens Healthcare, Erlangen, Germany). To quantify repeatability, the flow model was scanned three times at each center using both 2D PC MRI immediately followed by 4D Flow MRI. To quantify reproducibility, results were compared across the five centers. Agreement between 2D PC MRI and 4D Flow CSF velocity measurements were also quantified. Results were statistically analyzed within and across MRI centers and between measurement techniques using a linear mixed effects model.

### Subject specific in vitro csf flow model and experimental set-up

**Fig. 2:**
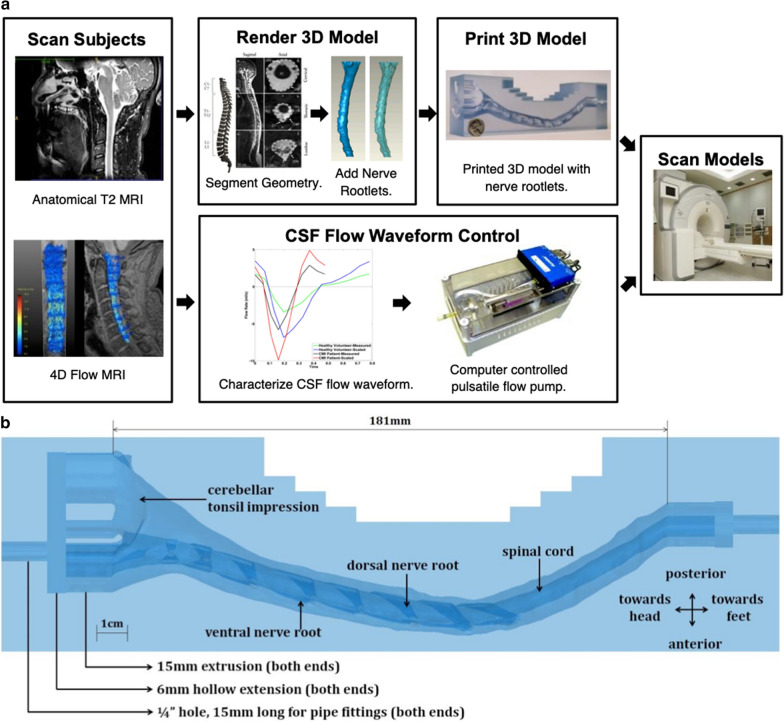
**a** Development of in vitro models based on subject specific scans. First, subjects were scanned to produce a T2 anatomical MRI and a 4D Flow MRI. The anatomical MRI was then used as a basis for the 3D model. The 4D Flow MRI allows the determination of the cerebrospinal fluid (CSF) flow waveform which informs a computer-controlled pump. The model is then connected to the pump and scanned at each MRI center. **b** Cross section of the completed model. A = anterior, P = posterior, S = superior, I = inferior

To control a consistent CSF flow waveform and anatomic shape across MRI measurement centers, we utilized a computer-controlled in vitro model CSF flow system previously developed by our research group [51] (Fig. 2a). The model was designed based on T2-weighted anatomical MRI data collected for a five-year-old Chiari malformation patient with 6.8 mm cerebellar tonsillar descent below the foramen magnum (FM), as described in Bunck et al. [14]. The spinal subarachnoid space was manually segmented from the medulla to the upper thoracic spine based on the T2-weighted images. Dorsal and ventral spinal cord nerve rootlets (NR) were added to the model segmentation based on ex-vivo anatomic measurements of nerve root location, radicular line, and descending angle. The model was printed by stereolithography with a spatial resolution of 75 µm (see Fig. 2b for model dimensions).

4D Flow images were acquired to quantify the subject-specific CSF flow waveform in the same Chiari malformation patient. CSF flow rate as a function of time was quantified based on a region of interest located at the C2–C3 vertebral level. This waveform was input to an in-house designed computer-controlled oscillatory syringe pump with pulse-trigger output (for MRI cardiac gating). To allow MRI scanning, the syringe output was connected to the in vitro models via polyethylene tubing. The pump was positioned outside of the scanner operating room with tubing connected to the in vitro model through the waveguide. Tubing was taped to the floor and scanner bed during operation to minimize tubing movement/vibrations during operation. Complete details on the in vitro system dimensions and characterization are provided by Thyagaraj et al. [51]. Scanning was repeated three times at each location. 4D Flow measurements from MRI machines are prone to eddy current offsets arising from non-uniformity of magnetic fields, therefore a static fluid body was placed next to the in vitro model during scanning for a post-processing eddy current offset correction. After affixing the static fluid bodies in place, each trial consisted of a 2D PC MRI scan immediately followed by a 4D Flow scan. Between subsequent trials, the model was manually repositioned by approximately a few centimeters within the scanner bed to mimic realistic conditions in clinics. This repositioning was to mimic the altered position that may occur if a human subject were to be re-scanned in the scanner bed with slightly different body orientation.

### In vitro imaging protocol

Imaging parameters were chosen to represent standard clinical procedures such that these results best represent the repeatability and reproducibility seen clinically.

4D Flow and 2D PC MRI images were collected at each center using the following settings (Table 3), adapted from a previous protocol [51]. We sought to have identical imaging parameters applied across all MRI machines and across the 4D Flow and 2D PC MRI protocol. In brief, 4D flow datasets were collected in the sagittal orientation with velocity encoding of 15 cm/s, prospective gating, 16 phases per cardiac cycle leading to a temporal resolution of 30 ms, repetition time (TR) of 7.5 ms, echo time (TE) of 4.6 ms, flip angle (FA) = 5°, with 1.5 mm isotropic resolution. Prospective gating of the model was based on the heart rate recorded in conjunction with the subject specific waveform collected for the computer-controlled model.

**Table 3:** MRI protocols used for 2D PC MRI and 4D Flow MRI acquisition

Parameter	2D PC MRI	4D Flow MRI
Spatial resolution	1.5 × 1.5 × 3	1.5 × 1.5 × 1.5
FOV [mm]	150 × 180	150 × 180x40
Number of heart phases	16	16
Parallel Imaging	No	2
Sym. Enc	Yes	Yes
Halfscan	No	0,75/1
Partial Echo	No	No
TR	5.5	7.5
TE	3.9	4.6
Flip angle	10	5
RF Spoiling	Yes	Yes
Scan Time	32 s	14 m35 s
BW	866	866
VENC	15	15
k-space segmentation factor	2	1
Trigger delay	7 ms	7 ms
Distortion correction	Yes	Yes

**Fig. 3:**
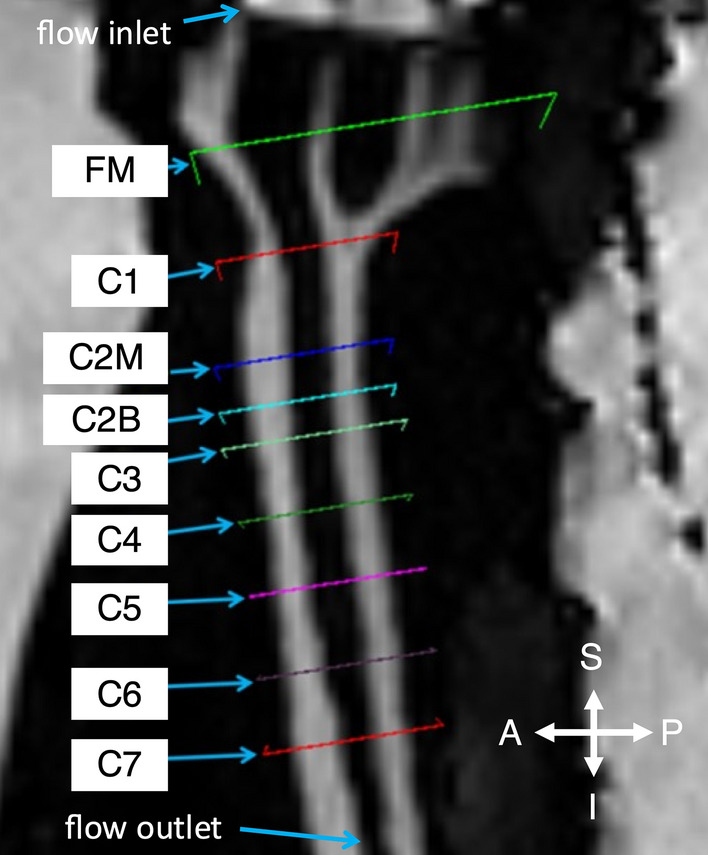
Axial positions of 2D PC MRI and 4D Flow MRI velocity measurements with flow inlets and outlets indicated. Distance between imaging planes can be found in Table 4. A = anterior, P = posterior, S = superior, I = inferior

2D PC MRI data was collected at nine axial slice positions along the model located as shown in Fig. 3 with distance between axial planes in Table 4. Total imaging time was approximately 15 min for the 4D Flow protocol and ~ 30 s for each 2D PC MRI scan. Slice positions relative to one another (i.e. foramen magnum to C1 vertebral level) was set to be identical across all MRI centers. The 4D Flow acquisition covered the entire region where 2D PC MRI slices were located.

**Table 4:** Distance of between axial planes imaged

Imaging plane	Distance from FM [mm]
FM	0
C1	12.7
C2M	25.7
C2B	31.2
C3	35.7
C4	44.7
C5	53.7
C6	63.7
C7	72.7

### MRI post-processing

Both 4D Flow and 2D PC MRI data were post-processed using GTFlow software (version 2.2.4, Gyrotools Inc, Zurich, Switzerland) by a single person at a center core lab. An eddy current offset correction was applied based on the static fluid body placed next to the in vitro model, to offset errors arising from non-uniformity of the magnetic field [52]. The flow field was also inspected and corrected for any aliasing artefacts when present. 2D PC MRI velocity data at each of the nine axial positions was exported as Matlab (version R2014b, Mathworks Inc, Natick, MA) readable files for quantitative comparison of CSF velocities. At each 2D PC MRI slice position, a 4D flow slice was selected and also exported to Matlab. To quantify peak systolic CSF velocity, first the phase corresponding to peak systole was identified using the maximum spatially averaged velocity of all pixels with non-zero velocities, defined as follows:

$$Spatial \, Average_{i} = \frac{{\sum\nolimits_{n = 1}^{N} {V_{n} } }}{N}$$

where *i* represents phase number, N represents total number of non-zero velocities, and V_n_ represent the thru-plane CSF velocity for the respective pixel. The peak systolic value was then measured as the pixel within the phase of peak systole having the greatest velocity value.

### Statistics

Because trial, scanning center, and scan type could have significant effects, we developed the following linear mixed-effects model for each replicate:

$$y = \beta_{0} + \mathop \sum \limits_{k = 1}^{13} \beta_{k} x_{k} + z_{1} + \mathop \sum \limits_{k = 2}^{13} z_{k} x_{k} + \varepsilon ,$$

where *y* is the velocity measurement along the spine, *x*s are binary covariates, *β*s are the fixed effects, and *z*s the random effects. Specifically, *x*_1_ indicates whether the treatment group is 4D Flow MRI or not, each of the *x*_*k*_s with *k*= 2,⋯,5 indicates whether the measurement was taken at the *j*th scanning center, and each of the *x*_*k*_s with *k*= 6,⋯,13 indicates whether the measurement was taken at one of the eight axial positions (C1, C2M, C2B, C3, C4, C5, C6, and C7). In this model, *β*_0_ represents the baseline, which is the mean velocity measurement from 2D PC MRI at scanning center #1 at the FM position along the spine. In other words, this model estimates the difference between another scanning center and Center 1, as well as between another axial position and FM. The baseline may also be the overall mean, or another center or axial position. Our analysis aims to test whether the regression coefficient is significantly different from 0; this is the same hypothesis no matter which baseline is chosen. Additionally, *z* represents random effects of the scanning centers and axial slice position (note that the treatment of 4D versus 2D is assumed to be a fixed effect and not included in the random effects), which follow a multivariate normal distribution with mean of zero and a symmetric variance–covariance matrix:

$${\varvec{z}}\sim N\left( {0,{{\varvec{\Sigma}}}} \right)$$

where *z* = (*z*_1_, ⋯, *z*_13_) is the column vector of all the random effects, 0 is a vector of zeros, and ∑ is the variance–covariance matrix. We used the Matlab (Ver. 2019a Mathworks Corp., Natick, MA) function “*fitlme*” to estimate the parameters in this linear mixed-effects model and test whether each of the fixed effect sizes is significantly different from zero. If so, this would indicate a statistically significant impact on the parameter from treatment groups, scanning centers, or axial position of velocity measurements.

Using this linear mixed-effects model, we obtained p-values for the following 14 fixed effect sizes: the baseline (Center 1), scan type (4D Flow MRI or not), scanning centers (Centers 2–5), axial position of measurement (C1, C2M, C2B, C3, C4, C5, C6, and C7). We accounted for multiple comparisons by applying the Bonferroni correction where the threshold for significant p-values was adjusted to be α/14, where α is the experimentwise type I error rate.

## Results

MR images were collected over three trials at five scanning centers using 2D PC MRI and 4D Flow. Trial 3 at Center 4 and trial 2 at Center 5 were excluded from analysis due to a bubble detected in the entrance tubing during scanning; all other scanning centers (Centers 1, 4, and 5) had three successful trials for each imaging modality that were included in analysis.

### Agreement of CSF velocity detection by 4D flow versus 2D PC MRI

**Fig. 4:**
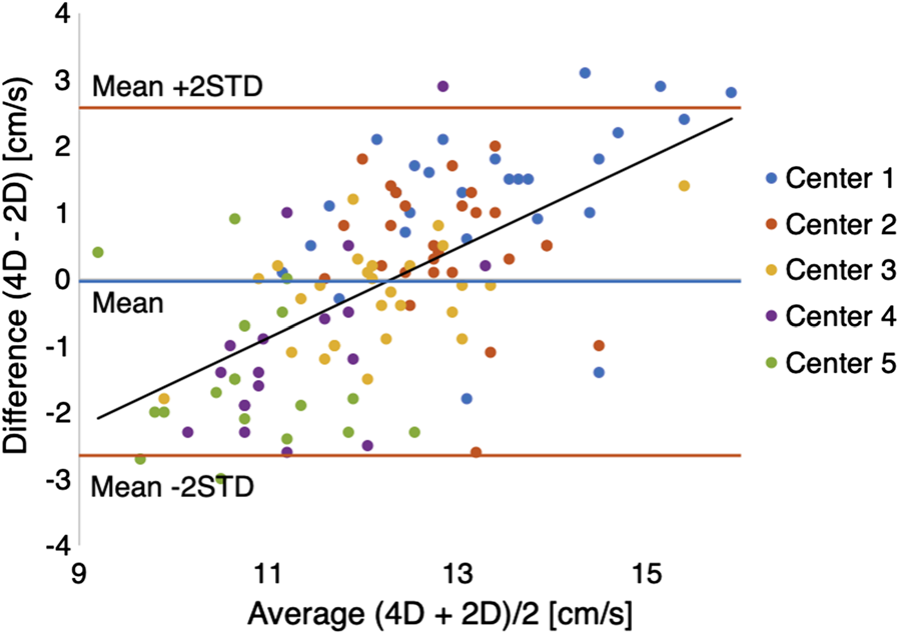
Bland–Altman plot showing agreement between 2D PC MRI and 4D Flow measurements; the trendline of the data is indicated by the black line, the mean of the differences is shown in blue, and the mean ± 2STD is indicated by the red lines

Our statistical analysis concluded that 4D Flow and 2D PC MRI are comparable methods for CSF velocity measurements at any scanning center and for any vertebral position. No evidence was found indicating disagreement (*p* = 0.86, Table 5) and there was moderate agreement seen in the Bland Altman Plot (Fig. 4). In all, there was an average difference of 0.02 cm/s between measurements of each scan type with a 95% confidence interval (CI) of −0.28 to 0.24 cm/s (Table 5) and a maximum difference of 2.9 cm/s (Fig. 4). No individual center had perfect agreement between 4D Flow and 2D PC MRI values. While the measurements showed no discernable trend relating to axial position of measurement, relative clusters formed for each scanning center showing that scanning center likely effects velocity measurement. Notably, a linear trend arose wherein the average velocity and difference between imaging modalities linearly decreased from Center 1 to Center 5, sequentially.

**Table 5:** Effect sizes and corresponding p values estimated from the linear mixed-effects model for velocity measurements

Effect	Effect size (95% CI) [cm/s]	p value
Intercept (Center 1: 2D PC MRI at FM)	14.16 (13.12, 15.21)	4.4 × 10^–74^*
Scan type (4D – 2D)	−0.02 (−0.28, 0.24)	0.86
Center 2	−0.45 (−1.27, 0.36)	0.27
Center 3	−1.17 (−2.63, 0.29)	0.12
Center 4	−2.09 (−3.42, 0.76)	2.2 × 10^–3^*
Center 5	−2.52 (−3.23, −1.76)	3.2 × 10^–10^*
C1	−0.80 (−1.37, −0.23)	0.006
C2M	−0.49 (−0.96, −0.02)	0.042
C2B	0.01 (−0.46, 0.48)	0.95
C3	−1.21 (−1.67, −0.75)	6.22 × 10^–7^*
C4	−1.36 (−1.85, −0.87)	1.08 × 10^–7^*
C5	−0.54 (−1.15, 0.07)	0.084
C6	−1.26 (−1.77, −0.75)	2.0 × 10^–6^*
C7	−1.71 (−2.17, −1.24)	9.5 × 10^–12^*

The mean effect size is provided, along with the 95% confidence interval (CI). We used Bonferroni correction to account for multiple testing. * represents statistical significance under Bonferroni correction where *p* < 0.05/14 = 0.0036

### Repeatability

**Fig. 5:**
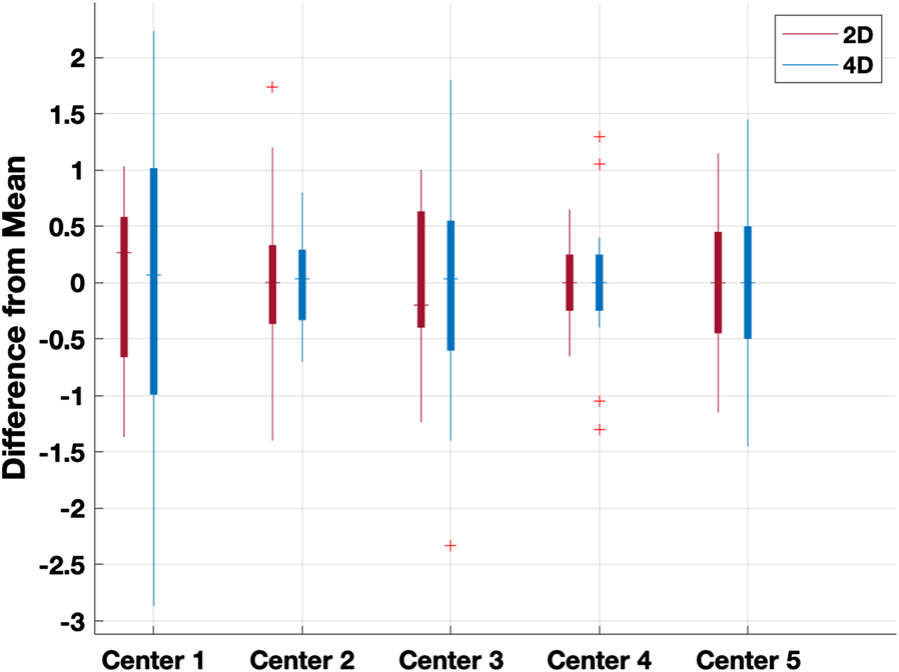
Box plot showing the difference between average velocity measurement at each axial location and each individual axial velocity measurements at each center. Top and bottom of boxes indicate 25th and 75th percentile of values with horizontal lines indicating the median of each value set and outliers represented as red cross marks

Repeatability within centers was relatively consistent with confidence intervals less than ± 2 cm/s (15% of the average measured value of 14 cm/s), (Fig. 5). 4D Flow and 2D PC MRI had similar degrees of repeatability, with some centers showing potentially more consistency of 2D PC MRI measurements and some showing better consistency of 4D Flow measurements. Comparatively, Center 2 showed the greatest degree of repeatability (STD = 0.87 cm/s, Table 6), Center 1 showed the worst degree of repeatability (STD = 1.50 cm/s, Table 6), and Centers 3–5 had relatively moderate repeatability (STD = 1.06 cm/s, 1.18 cm/s, and 1.25 cm/s, respectively, Table 6).

**Table 6:** Standard deviations of scanning centers for scanning type data subsets and over the entire dataset

	2D PC MRI [cm/s]	4D Flow [cm/s]	Overall [cm/s]
Center 1	1.13	1.58	1.50
Center 2	0.93	0.74	0.87
Center 3	1.00	1.22	1.06
Center 4	0.69	1.38	1.18
Center 5	1.08	0.92	1.25
Overall	1.04	1.83	1.49

### Reproducibility

**Fig. 6:**
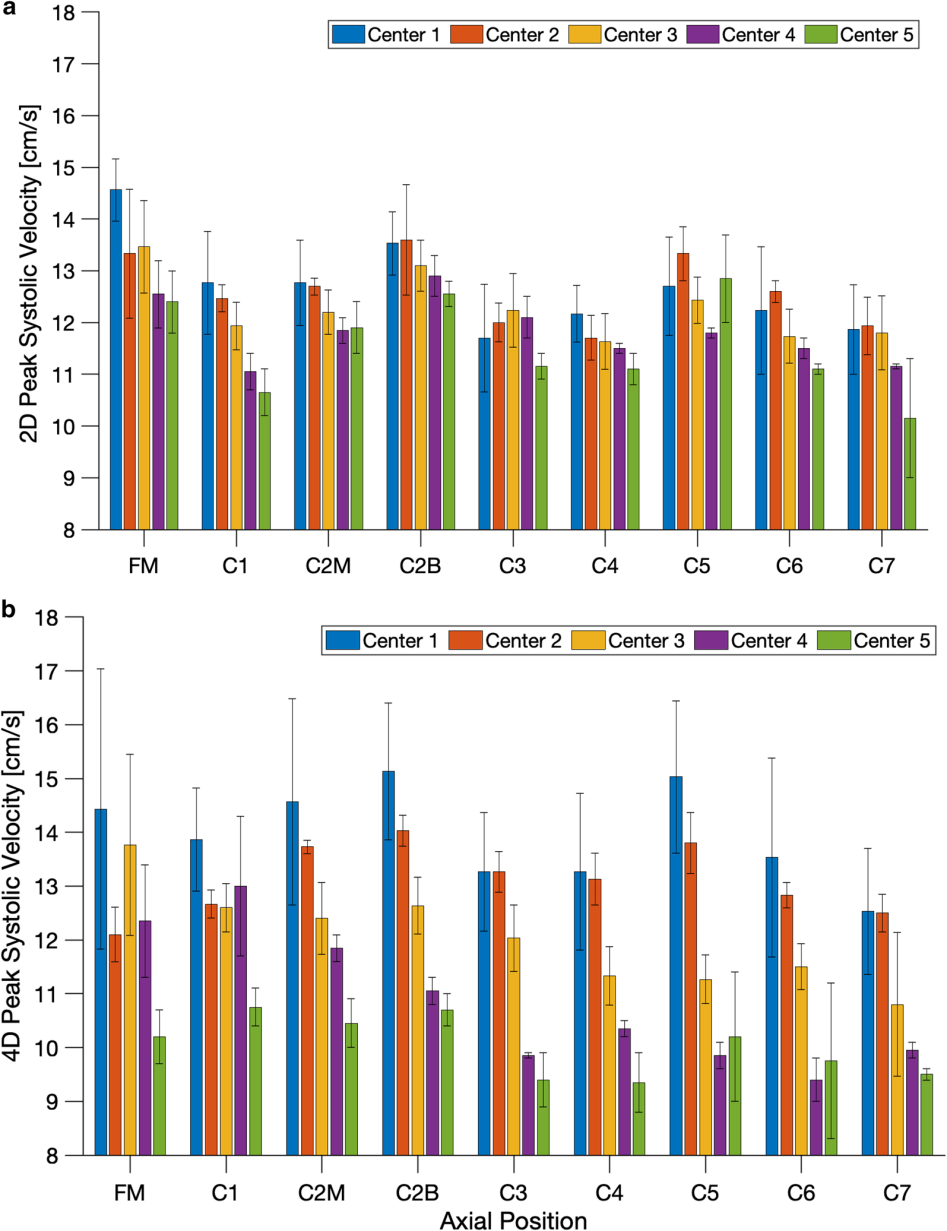
**a** Peak systolic 2D PC MRI CSF velocity at each axial position for each center. **b** Peak systolic 4D Flow CSF velocity at each axial position for each center. Error bars shown represent standard deviation

Peak systolic velocities lacked reproducibility across centers. Specifically, Center 2 (CI = −1.26, 0.36 cm/s; Table 5) and Center 3 (CI = −2.63, 0.29 cm/s; Table 5) were not significantly different from our baseline, Center 1, (Center 2: p = 0.27, Center 3: p = 0.12; Table 5) while Center 4 (CI = −3.42, −0.76 cm/s; Table 5) and Center 5 (CI = −3.23, −1.76 cm/s; Table 5) were statistically significantly different from baseline (Center 4: p = 2.2 × 10^–3^, Center 5: p = 3.3 × 10^–10^, Table 5). This lack of reproducibility can be seen in Fig. 6, wherein 4D Flow peak systolic velocity measurements displayed worse reproducibility than 2D PC MRI peak velocity measurements. Figure 4 also depicts this lack of reproducibility, as there is some overlap between Centers 1, 2, and 3 but Centers 4 and 5 are noticeably different. On average, peak systolic velocities at Center 1 were greater than Center 2 through 5, sequentially (Fig. 6). Each center appeared to have a relative offset value of measurements, indicating a calibration factor may be useful in future comparative studies of PC MRI measurement values.

## Discussion

This study quantifies agreement, repeatability, and reproducibility of 2D PC MRI and 4D Flow characterization techniques for the measurement of CSF flow velocities at the craniovertebral junction in Chiari malformation. We found that agreement between 2D PC MRI and 4D Flow was good, repeatability within any one scanner was fair, and reproducibility across centers was poor. An anatomically realistic in vitro CSF flow model was used to conduct experiments performed at five MRI scanning centers. Peak systolic velocities were found to range from 8.3 to 17.3 cm/s, which falls within the range of values reported in Chiari malformation patients (Table 1).

### Agreement

Peak systolic velocity values for 2D PC MRI and 4D Flow had overall good agreement for all centers analyzed with an average difference of 0.02 cm/s with 95% CI of −0.28 To 0.24 cm/s (Table 5). This finding supports that either technique can be used within a scanning center and the results would be comparable within exact slices. In clinical practice, a specific slice location is required for 2D PC MRI, while the slice location to be analyzed with 4D Flow is selected after image acquisition by re-slicing of the data. This provides added flexibility for analysis of CSF peak velocities that is not possible using 2D PC MRI. Our approach aimed to acquire 2D PC MRI and 4D Flow with similar spatial and temporal resolution (Table 3). However, it was not possible to identically match all scanner parameters which may have led to some differences in results across protocols.

Notably, variance across measurements was greater in 4D Flow results than 2D PC MRI (STD = 1.83 cm/s and 1.04 cm/s, respectively, Table 6). 4D Flow datasets seem to be closer to zero on average than the 2D PC MRI datasets yet the 2D PC MRI data has a narrower range of values than the 4D Flow data. That is to say, 2D PC MRI had less variance overall than 4D Flow across centers but failed to accurately estimate the mean as well as 4D Flow, therefore indicating greater precision and less accuracy in 2D PC MRI than 4D Flow measurements (Fig. 5). Without a “Gold Standard” known peak CSF velocity in the in vitro model, the underlying factor leading to this variance requires further research. This technique-based measurement variance can be seen in Fig. 4, where all measurements lie within ± 3 cm/s. Here, the overall good agreement between the techniques is apparent, but the relative clustering of values based on scanning center reveals an important insight into the reproducibility and repeatability of techniques. These center-based clusters could be due to scanner-specific effects at each center, wherein each scanner has a quantifiable effect on the measurements it makes. With a more focused research study, these scanner-effects can be understood and potentially mitigated by use of a standardized scanner calibration technique.

### Repeatability

Repeatability of measurement values within any scanner was dependent on each individual scanning center. This variance could be due to axial slice location relative to the model anatomy as peak velocity can vary significantly across the caudal brain and cervical spine. The difference in peak CSF velocity across axial positions was found to be significantly different from the foramen magnum (FM) baseline in four of eight locations (*p* < 0.05/14 = 0.0036, Table 5). Therefore, some variance is expected in the model and will likely be even greater in vivo. Figure 5 provides a visual depiction of the repeatability of either technique in each center where each measurement value was subtracted from the average peak systolic CSF velocity across axial positions for each center. Specifically, the horizontal bars across each box represent the median of each dataset; the closer this median bar is to zero, the better the repeatability within that center for that scanning technique.

### Reproducibility

Overall, the most important factor leading to measurement inconsistency in our study was lack of reproducibility across MRI scanning centers. Figure 6 shows that across axial positions, each center tended to have a relative offset based on the specific scanner used. In general, Center 1 reported the highest values for peak systolic CSF velocity followed by each other center sequentially, with Center 5 generally having the lowest reported peak systolic velocity values. This scanner-specific relative offset could be indicative of systemic difference across scanners. As mentioned above, this relative offset at each center is an important source of variance between scanning centers and could potentially be corrected by a standardized calibration procedure. This variance between scanning centers could potentially be due to scanner specific field inhomogeneity, eddy current generated during scanning, and/or inconsistency of the in vitro experimental set up. It is possible to eliminate eddy current offsets in 4D Flow scans by use of a zero-flow condition, whereby the resultant velocity field from that measurement can be applied for correction, but was not done here as this research is clinically oriented and sought to mimic in vivo conditions. Therefore, variance due to eddy current offsets during scanning are expected to be representative of those found in a clinical setting. We sought to reduce the effect of scanner specific field inhomogeneity with post-processing techniques, but it is to be expected in every clinical setting that there will be local magnetic field inhomogeneities and/or gradient imbalances that could be inconsistent over the whole field of view. To mitigate any potential experimental inconsistency, experiments were conducted with identical conditions across all centers including use of identical tubing, fittings, and computer controlled oscillatory pump and identical control waveform (see ”Methods”). Additional details on the in vitro system are also provided by Thyagaraj et al. [51]. Further, reproducibility varies slightly at different axial positions of imaging. This reproducibility is exaggerated at lower vertebral positions, with statistically significant differences at the C3, C4, C6, and C7 positions. Notably, C7 had the greatest significance (p = 9.5 × 10^–12^ < 0.05 /14 = 0.0036) and the largest effect size (−2.17, −1.24) of any vertebral position. At higher axial positions, specifically the FM–C2 levels, the difference is not significant. Therefore, we do not believe vertebral position contributed greatly to the lack of reproducibility.

### Case study—comparison of centers 2 and 3

Centers 2 and 3 utilized the same machine and provide an interesting case study, therefore a secondary statistics model was utilized wherein Center 2 was used as the reference rather than Center 1 (Additional file 1: Table S1). A statistically significant difference was found between Center 2 and Centers 4 and 5 (p = 1.4 × 10^–6^ and 4.1 × 10^–22^, respectively); no significant difference was found between Center 2 and Centers 1 and 3 (p = 0.27 and 0.11, respectively). These results show that while Centers 2 and 3 utilized the same machine and had a small amount of clustering (Fig. 4), Center 2 was most similar to Center 1, therefore utilizing the same type of machine does not guarantee how similar results will or will not be. Based on this, scanner calibration procedures should potentially be developed based on individual scanning machines.

### Relevance of findings to clinical diagnostics for chiari malformation

A meta-analysis of similar studies in literature and previous investigations of healthy and Chiari CSF dynamics reveal important insights for the clinical application of novel PC MRI peak velocity quantifications in the cervical spine. Figure 1 shows that these previous investigations of CSF dynamics reported consistently elevated peak CSF velocities in Chiari patients compared to healthy controls at every vertebral level, with a maximum difference of 6.9 cm/s at the C1 position. This difference points towards an underlying physiology of Chiari malformation at the C1 vertebral position that could be leveraged for improved diagnostics pending reliable detection, which requires disagreement between groups to be less than the effect size. Good agreement between 2D PC MRI and 4D Flow measurements indicates both methods would be acceptable in clinical use to characterize CSF dynamics. We also found intra-scanner repeatability of either measurement type to be good, but inter-scanner reproducibility was poor. This lack of reproducibility may help us understand previous studies with conflicting results regarding Chiari CSF dynamics. Mitigation of the lack of reproducibility across centers could be achieved with a standardized calibration procedure such as generating scanner specific reference values for healthy volunteers.

## Limitations

Several limitations have been identified within this study, the use of an in vitro model being the primary limitation. To better understand each parameter and its specific effects on measurement variability, a simple model with an analytical solution could be investigated in future studies with varying levels of complexity, though this was not done here as the focus of this research was for Chiari Malformation applications. We utilized an in vitro subject specific model of a pediatric Chiari patient, but in vivo studies are needed to understand the full range of physiologically-rooted variability that can occur such as the impact of respiration, movement artefacts, etc. The use of a pediatric Chiari patient for a subject specific model results in data that is not representative of all conditions and individual anatomies, limiting the application of these results to adult populations and other disease populations. The precise 3D flow field in the in vitro models has not been validated for any specific Chiari patient. This model used one representative flow waveform to control the oscillatory pump, which introduces further specificity of these results and limits a broader application. Importantly, the use of a rigid model here is not realistic to in vivo situations. Rigid model structures do not model tissue properties therefore flow field characteristics of such models cannot be accurately assessed and must be taken into consideration in the application of results shown here. Future studies to detail and develop an in vitro model of these tissue properties are necessary. Depending on the sensitivity of the measurements, the location of the imaging plane can cause error in the results and introduce an operator bias in the data. Operator dependence has been detailed by previous studies [45, 53] and therefore was not included here as a parameter of interest.

This study focused on measurement agreement, repeatability, and reproducibility and did not quantify a “Gold Standard” measurement for quantification of accuracy. While we sought to set up identical experiments at each center for each trial, the computer-controlled oscillatory pump can only control the waveform input to the inlet. Although tubing was relatively rigid, the exact waveform at the model outlet cannot be known without independent quantification. The exact waveform could be quantified by independent measurement of CSF velocities by flow measurement with laboratory bench-top devices but was outside the scope of this study. Finally, the use of computational flow dynamics could be used to further characterize velocity field errors and an accuracy based on the input flow waveform. Computational flow dynamics have been detailed in a study previously done by our group [34] and therefore were not included here.

## Conclusion

A patient-specific in vitro model of Type I Chiari malformation was used to quantify agreement, repeatability, and reproducibility of 2D PC MRI and 4D Flow quantification of peak CSF velocities. The single greatest factor leading to measurement inconsistency of peak CSF velocities was lack of inter-scanner reproducibility. Taken in combination, the results help identify sources of error that can be improved to allow better application of CSF velocity detection for medical diagnostic purposes. Overall, both 2D PC MRI and 4D Flow techniques show promise as diagnostic tools to quantify CSF dynamics in Chiari malformation.

## Supplementary Information

**Additional file 1: Figure S1A-D. **Forest Plot of meta-analysis separated by imaging modality (2D PC MRI vs 4D PC MRI) and Chiari vs healthy populations. **Table S1. **Effect sizes and corresponding p values estimated from the secondary linear mixed-effects model for velocity measurements. This model uses Center 2 as the baseline and includes interactions between scanning modality, scanning center, and axial position of imaging. The mean effect size is provided, along with the 95% confidence interval (CI). We used Bonferroni correction to account for multiple testing. * represents statistical significance under Bonferroni correction where the threshold is *p < *0.05/14=0.0036.

## Data Availability

The datasets used and/or analyzed during the current study are available from the corresponding author on reasonable request.

## Abbreviations

3D: Three-dimensional
CSF: Cerebrospinal fluid
DM: Dura matter
FM: Foramen magnum
FOV: Field of view
MR: Magnetic resonance
MRI: Magnetic resonance imaging
PC MRI: Phase contrast magnetic resonance imaging
PWV: Pulse wave velocity
SC: Spinal cord
SSS: Spinal subarachnoid space
TE: Echo time
TR: Repetition time

## References

[1] Lindstrom EK, Ringstad G, Mardal KA, Eide PK (2018). Cerebrospinal fluid volumetric net flow rate and direction in idiopathic normal pressure hydrocephalus. Neuroimage Clin.

[2] Blitz AM, Shin J, Baledent O, Page G, Bonham LW, Herzka DA, Moghekar AR, Rigamonti D (2018). Does phase-contrast imaging through the cerebral aqueduct predict the outcome of lumbar CSF drainage or shunt surgery in patients with suspected adult hydrocephalus?. AJNR Am J Neuroradiol.

[3] Martin BA, Kalata W, Shaffer N, Fischer P, Luciano M, Loth F (2013). Hydrodynamic and longitudinal impedance analysis of cerebrospinal fluid dynamics at the craniovertebral junction in type I Chiari malformation. PLoS ONE.

[4] Heiss JD, Snyder K, Peterson MM, Patronas NJ, Butman JA, Smith RK, Devroom HL, Sansur CA, Eskioglu E, Kammerer WA, Oldfield EH (2012). Pathophysiology of primary spinal syringomyelia. J Neurosurg Spine.

[5] Yeo J, Cheng S, Hemley S, Lee BB, Stoodley M, Bilston L (2017). Characteristics of CSF velocity-time profile in posttraumatic syringomyelia. AJNR Am J Neuroradiol.

[6] Shaffer N, Martin B, Loth F (2011). Cerebrospinal fluid hydrodynamics in type I Chiari malformation. Neurol Res.

[7] Bapuraj JR, Londy FJ, Delavari N, Maher CO, Garton HJL, Martin BA, Muraszko KM, Ibrahim EH, Quint DJ (2016). Cerebrospinal fluid velocity amplitudes within the cerebral aqueduct in healthy children and patients with Chiari I malformation. J Magn Reson Imaging.

[8] Korbecki A, Zimny A, Podgorski P, Sasiadek M, Bladowska J (2019). Imaging of cerebrospinal fluid flow: fundamentals, techniques, and clinical applications of phase-contrast magnetic resonance imaging. Pol J Radiol.

[9] Krueger KD, Haughton VM, Hetzel S (2010). Peak CSF velocities in patients with symptomatic and asymptomatic Chiari I malformation. AJNR Am J Neuroradiol.

[10] Geiger J, Markl M, Jung B, Grohmann J, Stiller B, Langer M, Arnold R (2011). 4D-MR flow analysis in patients after repair for tetralogy of Fallot. Eur Radiol.

[11] Barker AJ, Markl M, Burk J, Lorenz R, Bock J, Bauer S, Schulz-Menger J, von Knobelsdorff-Brenkenhoff F (2012). Bicuspid aortic valve is associated with altered wall shear stress in the ascending aorta. Circulation Cardiovasc Imag.

[12] Stadlbauer A, Salomonowitz E, van der Riet W, Buchfelder M, Ganslandt O (2010). Insight into the patterns of cerebrospinal fluid flow in the human ventricular system using MR velocity mapping. Neuroimage.

[13] Stankovic Z, Allen BD, Garcia J, Jarvis KB, Markl M (2014). 4D flow imaging with MRI. Cardiovasc Diagn Ther.

[14] Bunck AC, Kroeger JR, Juettner A, Brentrup A, Fiedler B, Crelier GR, Martin BA, Heindel W, Maintz D, Schwindt W, Niederstadt T (2012). Magnetic resonance 4D flow analysis of cerebrospinal fluid dynamics in Chiari I malformation with and without syringomyelia. Eur Radiol.

[15] Watts R, Steinklein JM, Waldman L, Zhou X, Filippi CG (2019). Measuring glymphatic flow in man using quantitative contrast-enhanced MRI. AJNR Am J Neuroradiol.

[16] Edeklev CS, Halvorsen M, Lovland G, Vatnehol SAS, Gjertsen O, Nedregaard B, Sletteberg R, Ringstad G, Eide PK (2019). Intrathecal use of gadobutrol for glymphatic MR imaging: prospective safety study of 100 patients. AJNR Am J Neuroradiol.

[17] Eide PK, Ringstad G (2019). Delayed clearance of cerebrospinal fluid tracer from entorhinal cortex in idiopathic normal pressure hydrocephalus: a glymphatic magnetic resonance imaging study. J Cereb Blood Flow Metab.

[18] Yildiz S, Thyagaraj S, Jin N, Zhong X, Heidari Pahlavian S, Martin BA, Loth F, Oshinski J, Sabra KG (2017). Quantifying the influence of respiration and cardiac pulsations on cerebrospinal fluid dynamics using real-time phase-contrast MRI. J Magn Reson Imaging.

[19] Chen L, Beckett A, Verma A, Feinberg DA (2015). Dynamics of respiratory and cardiac CSF motion revealed with real-time simultaneous multi-slice EPI velocity phase contrast imaging. Neuroimage.

[20] Aktas G, Kollmeier JM, Joseph AA, Merboldt KD, Ludwig HC, Gartner J, Frahm J, Dreha-Kulaczewski S (2019). Spinal CSF flow in response to forced thoracic and abdominal respiration. Fluids Barriers CNS.

[21] Dreha-Kulaczewski S, Konopka M, Joseph AA, Kollmeier J, Merboldt KD, Ludwig HC, Gartner J, Frahm J (2018). Respiration and the watershed of spinal CSF flow in humans. Sci Rep.

[22] Yamada S, Miyazaki M, Yamashita Y, Ouyang C, Yui M, Nakahashi M, Shimizu S, Aoki I, Morohoshi Y, McComb JG (2013). Influence of respiration on cerebrospinal fluid movement using magnetic resonance spin labeling. Fluids Barriers CNS.

[23] Yamada S, Goto T (2010). Understanding of cerebrospinal fluid hydrodynamics in idiopathic hydrocephalus (A) Visualization of CSF bulk flow with MRI time-spatial labeling pulse method (time-SLIP). Rinsho Shinkeigaku.

[24] Luciano MG, Batzdorf U, Kula RW, Rocque BG, Maher CO, Heiss J, Martin BA, Bolognese PA, Ashley-Koch A, Limbrick D (2019). Development of common data elements for use in Chiari Malformation type i clinical research: an NIH/NINDS project. Neurosurgery.

[25] Haughton VM, Korosec FR, Medow JE, Dolar MT, Iskandar BJ (2003). Peak systolic and diastolic CSF velocity in the foramen magnum in adult patients with Chiari I malformations and in normal control participants. Am J Neuroradiol.

[26] Quigley MF, Iskandar B, Quigley ME, Nicosia M, Haughton V (2004). Cerebrospinal fluid flow in foramen magnum: temporal and spatial patterns at MR imaging in volunteers and in patients with Chiari I malformation. Radiology.

[27] McGirt MJ, Atiba A, Attenello FJ, Wasserman BA, Datoo G, Gathinji M, Carson B, Weingart JD, Jallo GI (2008). Correlation of hindbrain CSF flow and outcome after surgical decompression for Chiari I malformation. Childs Nerv Syst.

[28] Sakas DE, Korfias SI, Wayte SC, Beale DJ, Papapetrou KP, Stranjalis GS, Whittaker KW, Whitwell HL (2005). Chiari malformation: CSF flow dynamics in the craniocervical junction and syrinx. Acta Neurochir (Wien).

[29] Thyagaraj S, Pahlavian SH, Sass LR, Loth F, Vatani M, Choi JW, Tubbs RS, Giese D, Kroger JR, Bunck AC, Martin BA (2017). An MRI-compatible hydrodynamic simulator of cerebrospinal fluid motion in the cervical spine. IEEE Trans Biomed Eng.

[30] Wentland AL, Grist TM, Wieben O (2013). Repeatability and internal consistency of abdominal 2D and 4D phase contrast MR flow measurements. Acad Radiol.

[31] Frydrychowicz A, Wieben O, Niespodzany E, Reeder SB, Johnson KM, Francois CJ (2013). Quantification of thoracic blood flow using volumetric magnetic resonance imaging with radial velocity encoding: in vivo validation. Invest Radiol.

[32] Drangova M, Zhu Y, Pelc NJ (1997). Effect of artifacts due to flowing blood on the reproducibility of phase-contrast measurements of myocardial motion. J Magn Reson Imaging.

[33] Sakhare AR, Barisano G, Pa J (2019). Assessing test-retest reliability of phase contrast MRI for measuring cerebrospinal fluid and cerebral blood flow dynamics. Magn Reson Med.

[34] Heidari Pahlavian S, Bunck AC, Thyagaraj S, Giese D, Loth F, Hedderich DM, Kroger JR, Martin BA (2016). Accuracy of 4D flow measurement of cerebrospinal fluid dynamics in the cervical spine: an in vitro verification against numerical simulation. Ann Biomed Eng.

[35] Meckel S, Leitner L, Bonati LH, Santini F, Schubert T, Stalder AF, Lyrer P, Markl M, Wetzel SG (2013). Intracranial artery velocity measurement using 4D PC MRI at 3 T: comparison with transcranial ultrasound techniques and 2D PC MRI. Neuroradiology.

[36] Feneis JF, Kyubwa E, Atianzar K, Cheng JY, Alley MT, Vasanawala SS, Demaria AN, Hsiao A (2018). 4D flow MRI quantification of mitral and tricuspid regurgitation: reproducibility and consistency relative to conventional MRI. J Magn Reson Imaging.

[37] Stalder AF, Russe MF, Frydrychowicz A, Bock J, Hennig J, Markl M (2008). Quantitative 2D and 3D phase contrast MRI: optimized analysis of blood flow and vessel wall parameters. Magn Reson Med.

[38] Gabbour M, Schnell S, Jarvis K, Robinson JD, Markl M, Rigsby CK (2015). 4-D flow magnetic resonance imaging: blood flow quantification compared to 2-D phase-contrast magnetic resonance imaging and Doppler echocardiography. Pediatr Radiol.

[39] Yzet T, Bouzerar R, Allart JD, Demuynck F, Legallais C, Robert B, Deramond H, Meyer ME, Baledent O (2010). Hepatic vascular flow measurements by phase contrast MRI and doppler echography: a comparative and reproducibility study. J Magn Reson Imaging.

[40] Alperin N, Hushek SG, Lee SH, Sivaramakrishnan A, Lichtor T (2005). MRI study of cerebral blood flow and CSF flow dynamics in an upright posture: the effect of posture on the intracranial compliance and pressure. Acta Neurochir Suppl.

[41] Alperin N, Loftus JR, Oliu CJ, Bagci AM, Lee SH, Ertl-Wagner B, Green B, Sekula R (2014). Magnetic resonance imaging measures of posterior cranial fossa morphology and cerebrospinal fluid physiology in Chiari malformation type I. Neurosurgery.

[42] Alperin N, Lee SH, Bagci AM (2015). MRI measurements of intracranial pressure in the upright posture: the effect of the hydrostatic pressure gradient. J Magn Reson Imaging.

[43] Tawfik AM, Elsorogy L, Abdelghaffar R, Naby AA, Elmenshawi I (2017). Phase-contrast MRI CSF flow measurements for the diagnosis of normal-pressure hydrocephalus: observer agreement of velocity versus volume parameters. AJR Am J Roentgenol.

[44] Luetmer PH, Huston J, Friedman JA, Dixon GR, Petersen RC, Jack CR, McClelland RL, Ebersold MJ (2002). Measurement of cerebrospinal fluid flow at the cerebral aqueduct by use of phase-contrast magnetic resonance imaging: technique validation and utility in diagnosing idiopathic normal pressure hydrocephalus. Neurosurgery.

[45] Martin BA, Yiallourou TI, Pahlavian SH, Thyagaraj S, Bunck AC, Loth F, Sheffer DB, Kroger JR, Stergiopulos N (2016). Inter-operator reliability of magnetic resonance image-based computational fluid dynamics prediction of cerebrospinal fluid motion in the cervical spine. Ann Biomed Eng.

[46] Yamada S, Tsuchiya K, Bradley WG, Law M, Winkler ML, Borzage MT, Miyazaki M, Kelly EJ, McComb JG (2015). Current and emerging MR imaging techniques for the diagnosis and management of CSF flow disorders: a review of phase-contrast and time-spatial labeling inversion pulse. AJNR Am J Neuroradiol.

[47] Yiallourou TI, Kroger JR, Stergiopulos N, Maintz D, Martin BA, Bunck AC (2012). Comparison of 4D phase-contrast MRI flow measurements to computational fluid dynamics simulations of cerebrospinal fluid motion in the cervical spine. PLoS ONE.

[48] Yamada S (2014). Cerebrospinal fluid physiology: visualization of cerebrospinal fluid dynamics using the magnetic resonance imaging Time-Spatial Inversion Pulse method. Croat Med J.

[49] Heidari Pahlavian S, Bunck AC, Loth F, Shane Tubbs R, Yiallourou T, Kroeger JR, Heindel W, Martin BA (2015). Characterization of the discrepancies between four-dimensional phase-contrast magnetic resonance imaging and in-silico simulations of cerebrospinal fluid dynamics. J Biomech Eng.

[50] Peters K, Weiss K, Maintz D, Giese D (2019). Influence of respiration-induced B0 variations in real-time phase-contrast echo planar imaging of the cervical cerebrospinal fluid. Magn Reson Med.

[51] Thyagaraj S, Pahlavian SH, Sass LR, Loth F, Vatani M, Choi JW, Tubbs RS, Giese D, Kroger JR, Bunck AC, Martin BA (2018). An MRI-compatible hydrodynamic simulator of cerebrospinal fluid motion in the cervical spine. IEEE Trans Biomed Eng.

[52] Walker PG, Cranney GB, Scheidegger MB, Waseleski G, Pohost GM, Yoganathan AP (1993). Semiautomated method for noise reduction and background phase error correction in MR phase velocity data. J Magn Reson Imaging.

[53] Glor FP, Long Q, Hughes AD, Augst AD, Ariff B, Thom SA, Verdonck PR, Xu XY (2003). Reproducibility study of magnetic resonance image-based computational fluid dynamics prediction of carotid bifurcation flow. Ann Biomed Eng.

[54] Bunck AC, Kroger JR, Juttner A, Brentrup A, Fiedler B, Schaarschmidt F, Crelier GR, Schwindt W, Heindel W, Niederstadt T, Maintz D (2011). Magnetic resonance 4D flow characteristics of cerebrospinal fluid at the craniocervical junction and the cervical spinal canal. Eur Radiol.

[55] Yiallourou TI, Kröger JR, Stergiopulos N, Maintz D, Martin BA, Bunck AC (2012). Comparison of 4D phase-contrast MRI flow measurements to computational fluid dynamics simulations of cerebrospinal fluid motion in the cervical spine. PLoS ONE.

[56] Shah S, Haughton V, del Rio AM (2011). CSF flow through the upper cervical spinal canal in Chiari I malformation. AJNR Am J Neuroradiol.

[57] Haughton VM, Korosec FR, Medow JE, Dolar MT, Iskandar BJ (2003). Peak systolic and diastolic CSF velocity in the foramen magnum in adult patients with Chiari I malformations and in normal control participants. AJNR Am J Neuroradiol.

[58] Dolar MT, Haughton VM, Iskandar BJ, Quigley M (2004). Effect of craniocervical decompression on peak CSF velocities in symptomatic patients with Chiari I malformation. AJNR Am J Neuroradiol.

[59] Hofmann E, Warmuth-Metz M, Bendszus M, Solymosi L (2000). Phase-contrast MR imaging of the cervical CSF and spinal cord: volumetric motion analysis in patients with Chiari I malformation. AJNR Am J Neuroradiol.

[60] Iskandar BJ, Quigley M, Haughton VM (2004). Foramen magnum cerebrospinal fluid flow characteristics in children with Chiari I malformation before and after craniocervical decompression. J Neurosurg.

[61] Rutkowska G, Haughton V, Linge S, Mardal KA (2012). Patient-specific 3D simulation of cyclic CSF flow at the craniocervical region. AJNR Am J Neuroradiol.

[62] Loth F, Yardimci MA, Alperin N (2001). Hydrodynamic modeling of cerebrospinal fluid motion within the spinal cavity. J Biomech Eng.

[63] Cheng S, Stoodley MA, Wong J, Hemley S, Fletcher DF, Bilston LE (2012). The presence of arachnoiditis affects the characteristics of CSF flow in the spinal subarachnoid space: a modelling study. J Biomech.

[64] Alperin N, Loftus JR, Bagci AM, Lee SH, Oliu CJ, Green BA: Magnetic resonance imaging-based measures predictive of short-term surgical outcome in patients with Chiari malformation Type I. J Neurol Neurosurg Psychiatry. 2015. 10.3171/2016.5.SPINE1621 27494782

[65] Koerte I, Haberl C, Schmidt M, Pomschar A, Lee S, Rapp P, Steffinger D, Tain RW, Alperin N, Ertl-Wagner B (2013). Inter- and intra-rater reliability of blood and cerebrospinal fluid flow quantification by phase-contrast MRI. J Magn Reson Imaging.

