# Supplementary material for: In vitro evaluation of cerebrospinal fluid velocity measurement in type I Chiari malformation: repeatability, reproducibility, and agreement using 2D phase contrast and 4D flow MRI
Source: Fluids Barriers CNS. 2021 Mar 18;18:12. doi: 10.1186/s12987-021-00246-3 (PMC7977612; doi:10.1186/s12987-021-00246-3)
Supplement: Supplementary file 1 — Additional file 1: Figure S1A-D. Forest Plot of meta-analysis separated by imaging modality (2D PC MRI vs 4D PC MRI) and Chiari vs healthy populations. Table S1. Effect sizes and corresponding p values estimated from the secondary linear mixed-effects model for velocity measurements. This model uses Center 2 as the baseline and includes interactions between scanning modality, scanning center, and axial position of imaging. The mean effect size is provided, along with the 95% confidence interval (CI). We used Bonferroni correction to account for multiple testing. * represents statistical significance under Bonferroni correction where the threshold is p < 0.05/14=0.0036. [file 12987_2021_246_MOESM1_ESM.pdf]

Supplementary Material.

Figure S1A-D. Forest Plot of meta-analysis separated by imaging modality (2D PC MRI vs 4D PC MRI) and Chiari vs healthy populations.

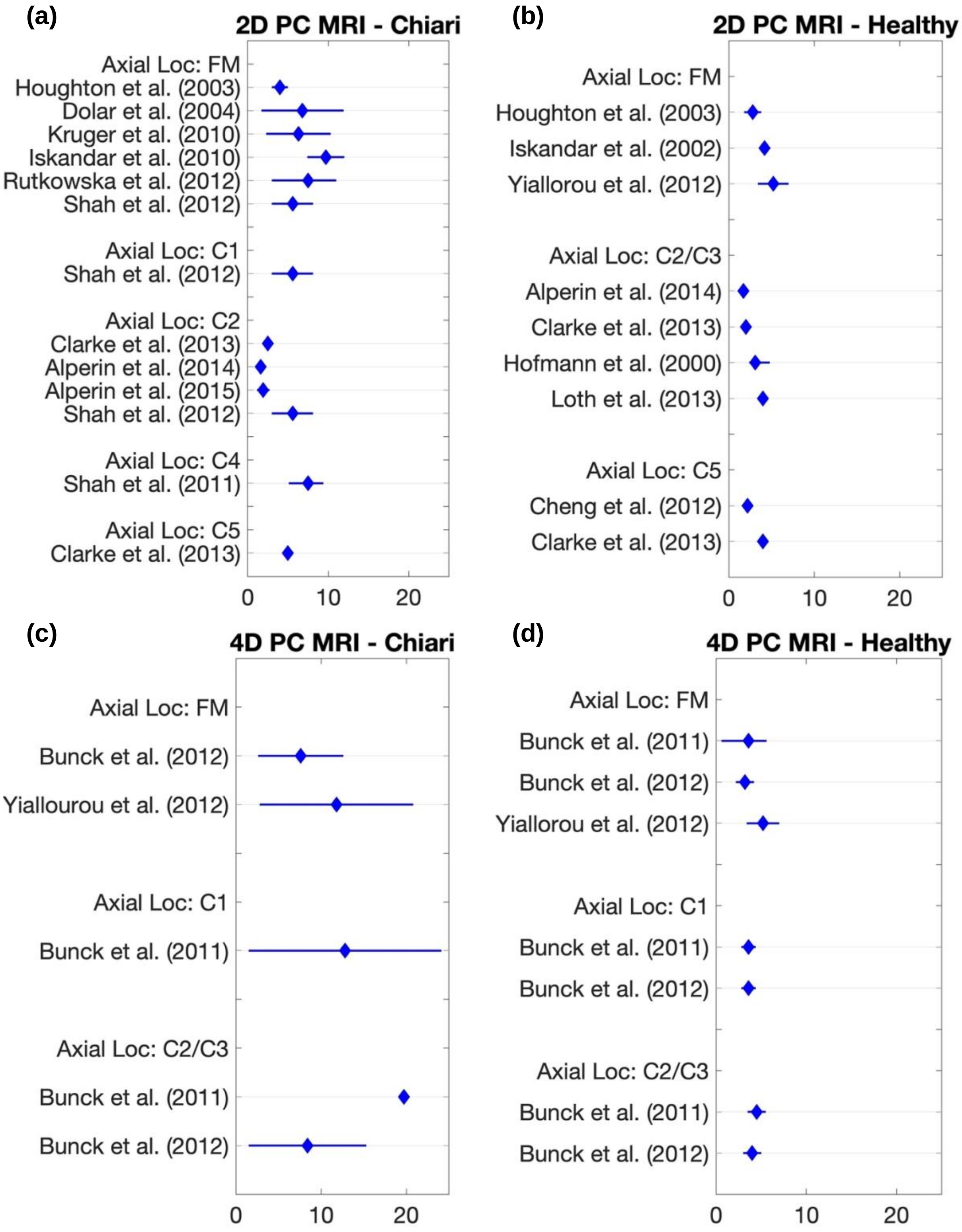

Table S1. Effect sizes and corresponding p values estimated from the secondary linear mixed-effects model for velocity measurements. This model uses Center 2 as the baseline and includes interactions between scanning modality, scanning center, and axial position of imaging. The mean effect size is provided, along with the 95% confidence interval (CI). We used Bonferroni correction to account for multiple testing. * represents statistical significance under Bonferroni correction where the threshold is $p < 0.05/14=0.0036$.

Effect	Effect Size (95% CI) [cm/s]	p value
Intercept (Center 2: 2D PC MRI at FM)	13.70 (13.27, 14.14)	4.57×10^{-141}*
Scan type (4D – 2D)	-0.02 (-0.28, 0.24)	0.86
Center 1	0.45 (-0.36, 1.27)	0.27
Center 3	-1.64 (-2.29, -0.99)	0.11
Center 4	-2.06 (-2.44, -1.69)	1.40×10^{-06}*
Center 5	-0.72 (-1.59, 0.15)	4.17×10^{-22}*
C1	-0.80 (-1.36, -0.24)	0.01
C2M	-0.49 (-0.96, -0.02)	0.04
C2B	0.01 (-0.45, 0.48)	0.95
C3	-1.21 (-1.67, -0.75)	5.78×10^{-07}*
C4	-1.36 (-1.85, -0.87)	1.01×10^{-07}*
C5	-0.54 (-1.14, 0.07)	0.08
C6	-1.26 (-1.77, -0.75)	1.89×10^{-06}*
C7	-1.71 (-2.17, -1.24)	8.37×10^{-12}
